# Phthisis Cured by Living on Raw Turtle

**Published:** 1831

**Authors:** 


### LXXIV.

### PHTHISIS CURED BY LIVING ON RAW TURTLE.

DR. HAMILTON, in his recent and elegantly-written History of Medicine, relates the following case as having come within his own observation in the West Indies.

"However the art of the cook may have succeeded in corrupting the natural qualities of the flesh of the turtle, by the addition of powerful condiments and inflammatory sauces, so as to bring upon it an unmerited stigma; in its unsophisticated state, it is perhaps one of the most nutritious, salutary, and restorative, articles of diet in existence. It was most satisfactorily proved in a case which fell but a few years since under the observation of the writer of this volume, and can nowhere find a place more appropriate for its introduction than the present. A gentleman, resident in one of our oldest and most frequented islands in the West Indies, having, from a long continued course of free living and late hours, contracted a complaint, to all appearance confirmed phthisis, accompanied with excessive emaciation, debility, teasing hectic cough, and all the other ordinary symptoms—when wasted to but a shadow of his former self, was advised, as a last, though almost hopeless, resource, to try change of air, and endeavour to glean health from the sanative breezes of the uninhabited island of Testigos, on the coast of the Spanish main. This is a low sandy island, destitute of human habitations, and only resorted to at particular seasons by fishermen, who come in quest of the turtle, which frequent its shores, unintimidated by the sight of man. Testigos could thus boast no vintners' cellars or luxurious taverns, to debauch the appetite and sap the constitution—no social parties to tempt to indulgence—no boon companions to betray into intemperance: nature alone presided within its solitary bounds, and compelled obedience, however reluctant, to her salutary laws. So deplorable was the condition of the invalid, and so utterly hopeless appeared to be his chance of recovery, that, on embarking for that island, which he hardly expected to reach alive, he carried a supply of planks and tools along with him, for the double purpose of constructing a hut to shelter him in life, and afterwards form a coffin to receive him in death. Arrived at Testigos, he led from necessity a life of primæval regularity and simplicity, rising with the orb of day, and courting the balmy influence of sleep, as soon as

---

'Implore the passing tribute of a sigh,'

from every one who has a spark of feeling in the heart."—*See View from the Tower of the Capitol, p.* 160.

the shades of evening fell—his food was the turtle's flesh, uncontaminated by the arts of the cook, which tempt the palate but undermine the health—his drink the simple element, uninflamed by the admixture of deceitful wine or inebriating spirit. After thus living in patriarchal simplicity for some few months, he returned to his astonished friends, so altered in appearance, and so renovated in health, that even those who had been most intimate with him hardly knew him upon his arrival, and could with difficulty be persuaded that the robust and ruddy-complexioned being before them, was the same who had so recently parted from them a living skeleton—a wasted form." *V.* I. *p.* 350.

With all due deference for the learned author of the work above-mentioned and of the case just quoted, we suspect that the old West Indian laboured under a disease very different from phthisis. That the primæval simplicity of regimen pursued on the island of Testigos effected a cure in the case in question, we do not doubt; but we think the disease was seated in organs below the diaphragm.

---

